# Characterisation of genes encoding for extended spectrum β-lactamase in Gram-negative bacteria causing healthcare-associated infections in Mwanza, Tanzania

**DOI:** 10.4102/ajlm.v12i1.2107

**Published:** 2023-04-12

**Authors:** Jenipher G. Mwakyabala, Conjester I. Mtemisika, Stacy Mshana, Adam A. Mwakyoma, Vitus Silago

**Affiliations:** 1 Department of Microbiology and Immunology, Weill Bugando School of Medicine, Catholic University of Health and Allied Sciences, Mwanza, United Republic of Tanzania; 2 Molecular Biology Laboratory, Central Pathology Laboratory, Bugando Medical Centre, Mwanza, United Republic of Tanzania; 3 Department of Clinical Microbiology, Kilimanjaro Christian Medical Centre, Moshi, United Republic of Tanzania

**Keywords:** beta lactamases, extended spectrum beta-lactamase, Gram-negative bacteria, healthcare-associated infections, multiplex PCR assay

## Abstract

Healthcare-associated infections (HCAIs) caused by extended spectrum β-lactamase-producing Gram-negative bacteria (ESBL-GNB) increase morbidity and mortality. This cross-sectional study characterised ESBL genes (*bla*_CTX-M_, *bla*_TEM_ and *bla*_SHV_) among 30 ceftriaxone-resistant GNB causing HCAIs between January 2022 and July 2022 by multiplex polymerase chain reaction assay at the zonal referral hospital in Mwanza, Tanzania. Twenty-five (83.3%) had at least one ESBL gene, of which 23/25 (92.0%) carried the *bla*_CTX-M_ gene. Seventy-two percent (18/25) of the GNB-ESBL isolates carried more than one ESBL gene, of which the majority (88.8%; *n* = 16/25) carried the *bla*_CTX-M_ and *bla*_TEM_ genes. Extended spectrum β-lactamase genes, particularly *bla*_CTX-M_, are common among ceftriaxone-resistant GNB causing HCAIs.

**What this study adds:**

This study revealed the distribution of genes (*bla*_CTX-M_, *bla*_TEM_ and *bla*_SHV_) coding for ESBL production among ceftriaxone resistant GNB causing HCAIs However, all ESBL producing GNB were susceptible towards ceftriaxone-sulbactam indicating that ceftriaxone-sulbactam may be empirically prescribed for treating patients with HCAIs.

## Introduction

Healthcare-associated infections (HCAIs), also referred to as nosocomial infections, are infections acquired by patients while receiving healthcare services from ≥ 48 h after admission to a healthcare facility.^1,2,3^ Admission into intensive care units increases the risk of acquiring HCAIs due to (1) chronic diseases which lower body immunity; (2) surgical procedures which interfere with natural body defenses; and (3) medical invasive devices such as urinary catheters, central lines and intubators, which provide bacteria with direct entry into bodily tissues.^4^ *Escherichia coli, Klebsiella aerogenes, Enterobacter* spp., *Acinetobacter baumannii* and *Pseudomonas aeruginosa* are the most common Gram-negative bacteria (GNB) known to cause HCAIs.^5,6,7,8^

Healthcare-associated infections are associated with significant increased cost of healthcare services, days of hospitalisation and mortality.^9^ Healthcare-associated infections caused by multidrug-resistant bacteria phenotypes, such as extended spectrum β-lactamase-producing GNB (ESBL-GNB), further exaggerate morbidity and mortality. At the study site in Mwanza, Tanzania, the prevalence of HCAIs in surgical site infections ranges from 10% to 26%.^5,9,10^ *Staphylococcus aureus* accounts for nearly one-third of these, of which about 16% to 19% are methicillin resistant.^5,9^ On the other hand, only one study reported 13% of implicating GNB showed ESBL phenotypes.^9^ To date, the distribution of ESBL genes among ESBL-GNB phenotypes causing HCAIs is not clearly known. This study unravels the distributions of ESBL genes (*bla*_CTX-M_, *bla*_TEM_ and *bla*_SHV_) among ceftriaxone-resistant GNB causing HCAIs at a zonal referral hospital in Mwanza, Tanzania.

## Methods

### Ethical considerations

This study received ethical approval from the joint Catholic University of Health and Allied Sciences and Bugando Medical Centre Research Ethics and Review Committee. The study approval number is CREC: 2368/2022. All participants voluntarily provided written informed consent before being enrolled in the study. Unique identification numbers were used to ensure confidentiality. Laboratory results were communicated in a timely manner to attending doctors in order to guide rational therapy.

### Study design, population, setting and duration

This was a cross-sectional laboratory-based study of ceftriaxone-resistant GNB isolated from different HCAIs between January 2022 and July 2022 (unpublished data) at Bugando Medical Centre – a zonal referral hospital located in Mwanza, Tanzania. The bacterial isolates, which had been archived in 20% glycerol stocks stored in a –40 °C freezer in the Microbiology laboratory as part of a biorepository, were recovered for this study in July 2022. The duration of archive ranged from 1 to 6 months before recovery for molecular characterisation of ESBL genes. Clinical information related to each isolate, namely ward or clinic of origin, sample type, bacterial species name, and susceptibility towards third-generation cephalosporins, notably ceftriaxone, was extracted from the laboratory database. Laboratory procedures were conducted in Microbiology Research Laboratory and Molecular Biology Research Laboratory at the Catholic University of Health and Allied Sciences located at Bugando Medical Centre in Mwanza, Tanzania.

### Definition of healthcare-associated infection

In the current study, HCAI was defined as an infection that a patient develops after 48 h of hospital admission, while receiving healthcare for another disease or condition.^11^

### Laboratory procedure

#### Recovery of CRO-R-GNB causing healthcare-associated infections and phenotypic detection of ESBL production

Ceftriaxone-resistant GNB causing HCAIs were recovered by sub-culturing on plates of MacConkey agar with salt (MCA; HiMedia, Mumbai, India). Plates were incubated aerobically at 35 °C ± 2 °C for 20 h – 24 h followed by phenotypic detection of ESBL production and DNA extraction for multiplex polymerase chain reaction (PCR) assay. The disk combination method (DCM) from the Clinical and Laboratory Standards Institute^12^ was used for phenotypic detection of ESBL production among recovered ceftriaxone-resistant GNB.

#### DNA extraction

From 5 to 10 fresh colonies (≤ 24 h) of ceftriaxone-resistant GNB on plates of MCA were used for DNA extraction. A protocol for DNA extraction from GNB by QIAmp Min DNA extraction kit (QIAGEN, Wuerzburg, Germany) was used according to manufacturer’s instructions. DNA samples were stored at −20 °C.

#### Multiplex PCR assay

A multiplex PCR assay described by Monstein et al.^13^ was used for amplification and detection of ESBL genes (*bla*_CTX-M_, *bla*_SHV_, and *bla*_TEM_). Briefly, 2 µL of each DNA sample was added into a PCR reaction tube containing HotStarTaq DNA polymerase master mix (New England Biolabs; Hitchin, Hertfordshire, United Kingdom) and a set of primers (Table 1), resulting in a final PCR reaction volume of 25 µL. The thermal cycler (T100™, BIO-RAD, Kaki-Bukit, Singapore) was run with the following conditions: initial denaturation at 95 °C for 5 min; 30 cycles of denaturation at 94 °C for 30 s, annealing at 56 °C for 30 s, and extension at 72 °C for 1 min; and a final extension at 72 °C for 10 min. Products were detected by using a 1% agarose gel with Tris-acetate-EDTA buffer stained with SafeView^TM^ DNA stain (ABM; Richmond, British Colombia, Canada) and visualised under ultraviolet light.

**TABLE 1:** Sequences of primers used for multiplex polymerase chain reaction assays for extended spectrum β-lactamase genes, Bugando Medical Centre, Mwanza, Tanzania, January 2022 – July 2022.

Primer	Sequence (5ʹ-3ʹ direction)	Amplicon size	Reference
*bla*-SHV.SE forward	ATGCGTTATATTCGCCTGTG	747 bp	Monstein et al.^13^
*bla*-SHV.AS reverse	TGCTTTGTTATTCGGGCCAA		
TEM-164.SE forward	TCGCCGCATACACTATTCTCAGAATGA	445 bp	
TEM-165.AS reverse	ACGCTCACCGGCTCCAGATTTAT		
CTX-M-U1 forward	ATGTGCAGYACCAGTAARGTKATGGC	593 bp	
CTX-M-U2 reverse	TGGGTRAARTARGTSACCAGAAYCAGCGG		

*Source*: Adapted from Monstein HJ, Ostholm-Balkhed A, Nilsson MV, Nilsson M, Dornbusch K, Nilsson LE. Multiplex PCR amplification assay for the detection of blaSHV, blaTEM and blaCTX-M genes in Enterobacteriaceae. APMIS. 2007;115(12):1400–1408. https://doi.org/10.1111/j.1600-0463.2007.00722.x

bp, base pairs.

### Data management and analysis

Quantitative data were descriptively analysed by using Microsoft Excel (Microsoft Office; Redmond, Washington, United States) and Stata version 15.0 (StataCorp LLP; College Station, Texas, United States; https://www.stata.com/stata15/).

## Results

**FIGURE 1:**
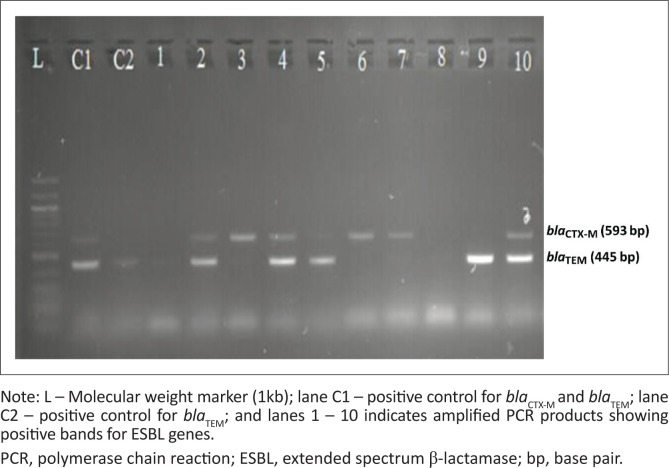
Molecular characterisation of extended spectrum β-lactamase genes by multiplex polymerase chain reaction assay, Bugando Medical Centre, Mwanza, Tanzania, January 2022 – July 2022.

A total of 30 ceftriaxone-resistant GNB causing HCAIs were recovered during this study period. Most of the recovered bacteria were *E. coli* 43.3% (*n* = 13). The majority of ceftriaxone-resistant GNB were isolated from the burn unit (40%; *n* = 12), and from pus/pus swab samples (56.6%; *n* = 17). By DCM, all (100%; *n* = 30) ceftriaxone-resistant GNB had positive ESBL phenotypes. Multiplex PCR assay revealed that about 83.3% (*n* = 25) had at least one ESBL gene, of which the majority (92.0%; *n* = 23) harboured the *bla*_CTX-M_ gene. Out of 25 GNB carrying ESBL genes, 18 (72.0%) carried multiple genes; of these, 88.8% (*n* = 16) carried *bla*_CTX-M_ and *bla*_TEM_ genes (Table 2 and Figure 1). Five isolates with negative PCR were *E. coli* (*n* = 3), isolated from pus/pus swab samples in the burn unit, and *Acinetobacter* spp. (*n* = 2), one isolated from a urine sample in the medical ward and the other isolated from a pus/pus swab sample from the neonatal intensive care unit (Table 3).

**TABLE 2:** Description of ceftriaxone-resistant GNB recovered for multiplex polymerase chain reaction amplification and detection of extended spectrum β-lactamase genes, Bugando Medical Centre, Mwanza, Tanzania, January 2022 – July 2022.

Variable	Frequency (*n*)	Percentage (%)
**Ward/unit of isolation**		
Burn unit	12	40.0
NICU	7	23.3
PICU	5	16.6
Medical ward	4	13.3
AICU	2	6.6
**Sample of origin**		
Pus or pus swab	17	56.6
Urine	7	23.3
Blood	6	19.9
**Isolate name**		
*E. coli*	13	43.3
*K. aerogenes*	7	23.3
*E. cloacae*	3	10.0
*Acinetobacter* spp.	3	10.0
*P. aeruginosa*	2	6.6
*K. oxytoca*	1	3.3
*A. hydrophilia*	1	3.3
**Disk combination method results**		
Positive	30	100.0
Negative	0	0.0
**Multiplex PCR amplification results**		
Positive	25	83.3
Negative	5	16.7
**Type of ESBL gene**		
*bla*_CTX-M_	23	92.0
*bla*_TEM_	18	64.2
*bla*_SHV_	2	8.0
**Multiple ESBL genes**		
Yes	18	72.0
No	1	28.0
**Type of multiple ESBL genes**		
*bla*_CTX-M_ and *bla*_TEM_	16	88.8
*bla*_CTX-M_ and *bla*_SHV_	1	5.6
*bla*_CTX-M_ and *bla*_TEM_ and *bla*_SHV_	1	5.6

AICU, adult intensive care unit; NICU, neonatal intensive care unit; PICU, paediatric intensive care unit; ESBL, extended spectrum β-lactamase; PCR, polymerase chain reaction.

**TABLE 3:** Results of disk combination method and multiplex PCR assay, and distributions of ESBL genes, Bugando Medical Centre, Mwanza, Tanzania, January 2022 – July 2022.

Isolate ID	Ward/unit	Sample	Species name	DCM	PCR	ESBL family		
						SHV	TEM	CTX-M
013HCAI	AICU	Blood	*K. aerogenes*	Pos	Pos	-	+	+
098HCAI	AICU	Urine	*E. coli*	Pos	Pos	-	+	-
001HCAI	Burn unit	Pus	*Acinetobacter spp.*	Pos	Pos	-	-	+
002HCAI	Burn unit	Pus	*E. coli*	Pos	Pos	-	+	+
004HCAI	Burn unit	Pus	*K. aerogenes*	Pos	Pos	-	+	+
009HCAI	Burn unit	Pus	*E. cloacae*	Pos	Pos	-	+	+
010HCAI	Burn unit	Pus	*K. aerogenes*	Pos	Pos	-	+	+
051HCAI	Burn unit	Pus	*E. coli*	Pos	Pos	-	+	+
062HCAI	Burn unit	Pus	*E. coli*	Pos	Neg	-	-	-
063HCAI	Burn unit	Pus	*E. coli*	Pos	Pos	-	+	+
071HCAI	Burn unit	Pus	*E. coli*	Pos	Neg	-	-	-
092HCAI	Burn unit	Pus	*P. aeruginosa*	Pos	Pos	-	-	+
093HCAI	Burn unit	Pus	*A. hydrophila*	Pos	Pos	-	-	+
094HCAI	Burn unit	Pus	*E. coli*	Pos	Neg	-	-	-
04HCAI-1	Medical ward	Urine	*E. coli*	Pos	Pos	-	+	+
04HCAI-2	Medical ward	Urine	*P. aeruginosa*	Pos	Pos	-	+	+
08HCAI	Medical ward	Urine	*Acinetobacter spp.*	Pos	Neg	-	-	-
020HCAI	Medical ward	Urine	*E. coli*	Pos	Pos	-	+	+
014HCAI	NICU	Pus	*Acinetobacter spp.*	Pos	Neg	-	-	-
021HCAI-1	NICU	Pus	*E. coli*	Pos	Pos	-	+	+
021HCAI-2	NICU	Pus	*K. aerogenes*	Pos	Pos	-	-	+
036HCAI	NICU	Pus	*K. aerogenes*	Pos	Pos	-	-	+
081HCAI	NICU	Blood	*E. cloacae*	Pos	Pos	-	+	-
082HCAI	NICU	Blood	*E. cloacae*	Pos	Pos	+	+	+
091HCAI	NICU	Pus	*K. aerogenes*	Pos	Pos	-	+	+
020HCAI	PICU	Blood	*E. coli*	Pos	Pos	-	-	+
077HCAI	PICU	Blood	*E. coli*	Pos	Pos	-	+	+
079HCAI-1	PICU	Urine	*E. coli*	Pos	Pos	-	+	+
079HCAI-2	PICU	Urine	*K. oxytoca*	Pos	Pos	+	-	+
086HCAI	PICU	Blood	*K. aerogenes*	Pos	Pos	-	+	+

HCAI, healthcare-associated infection; AICU, adult intensive care unit; NICU, neonatal intensive care unit; PICU, paediatric intensive care unit; DCM, disk combination method; ESBL, extended spectrum β-lactamase; SHV, sulfhydryl reagent variable beta-lactamase; TEM, temoniera beta-lactamase; CTX-M, cefotaximase-Munich β-lactamase; PCR, polymerase chain reaction; Pos, positive; Neg, negative.

## Discussion

The current study characterised the proportions and distributions of ESBL genes (*bla*_CTX-M_, *bla*_TEM_, and *bla*_SHV_) among ceftriaxone-resistant GNB which were isolated from different HCAIs between January 2022 and July 2022 at a tertiary zonal referral hospital in Mwanza, Tanzania. The majority of ceftriaxone-resistant GNB were recovered from the burn unit, from patients with burn injuries who were prone to infections because of the breached skin barrier.^14^ Moreover, *E. coli* accounted for the majority of recovered bacterial species, suggesting the patients’ own gut flora as an endogenous source of infection.^3^ However, *E. coli* can also be acquired from exogenous sources, such as contaminated inanimate surfaces, whenever hospital environmental cleaning and decontamination are poor.^15^

This study observed that all ceftriaxone-resistant GNB had positive ESBL phenotypes by DCM, even though four out of five (83.3%) ESBL phenotypes had at least one ESBL gene on multiplex PCR assay. Our findings are similar to a study by Silago et al., conducted in Mwanza, Tanzania, in 2020, which reported a proportion of 93.3% ESBL among GNB isolated from the hospital environment and hospitalised patients at the same setting.^16^ Our findings are, however, higher than a study by Said et al., which was conducted in 2021 in Mwanza, Tanzania, which reported that about 65.9% of GNB colonising children, of whom the majority were not hospitalised, harboured ESBL genes at the same setting.^17^ Therefore, the difference in study populations between the studies may explain the difference observed. Similar to previous studies published in 2020 and 2021 in Mwanza and in 2021 in Morogoro, Tanzania,^16,17,18^ the majority of ceftriaxone-resistant GNB were harbouring the *bla*_CTX-M_ gene. The predominance of *bla*_CTX-M_ may be a result of successful dissemination by conjugative epidemic plasmids, which facilitates its horizontal and vertical transmission.^16,19,20,21^

Five confirmed ESBL phenotypes by DCM did not harbour any of the three ESBL genes (*bla*_CTX-M_, *bla*_TEM_, and *bla*_SHV_) by multiplex PCR assay. This observation is in line with previous studies conducted from the same setting, Mwanza, Tanzania, in 2020 and 2021.^16,17^ The isolates may be harbouring other ESBL families which are non-cefotaximase-Munich beta-lactamase (non-CTX-M), non-temoniera beta-lactamase (non-TEM), and non-sulfhydryl reagent variable beta-lactamase (non-SHV), such as oxacillinase beta-lactamase, *Pseudomonas* extended resistant, Vietnam extended-spectrum β-lactamase, Tlahuica Indian- and Guiana-extended spectrum families.^22^

### Limitations

The small sample size of ceftriaxone-resistant GNB isolates obtained for this study is a weakness but did not affect the interpretation of the results.

### Conclusion

Extended spectrum β-lactamase genes, to be specific *bla*_CTX-M_, are common among ceftriaxone-resistant GNB causing HCAIs. Therefore, rational management of patients with HCAIs, guided by culture and sensitivity, is warranted.
